# Supplementary material for: The Construction and Exploration of a Comprehensive MicroRNA Centered Regulatory Network in Foxtail Millet (Setaria italica L.)
Source: Front Plant Sci. 2022 May 6;13:848474. doi: 10.3389/fpls.2022.848474 (PMC9121102; doi:10.3389/fpls.2022.848474)
Supplement: Supplementary file 3 [file Data_Sheet_3.zip › Sit-miRN2213_Seita.8G000100.1_353_TPlot.pdf]

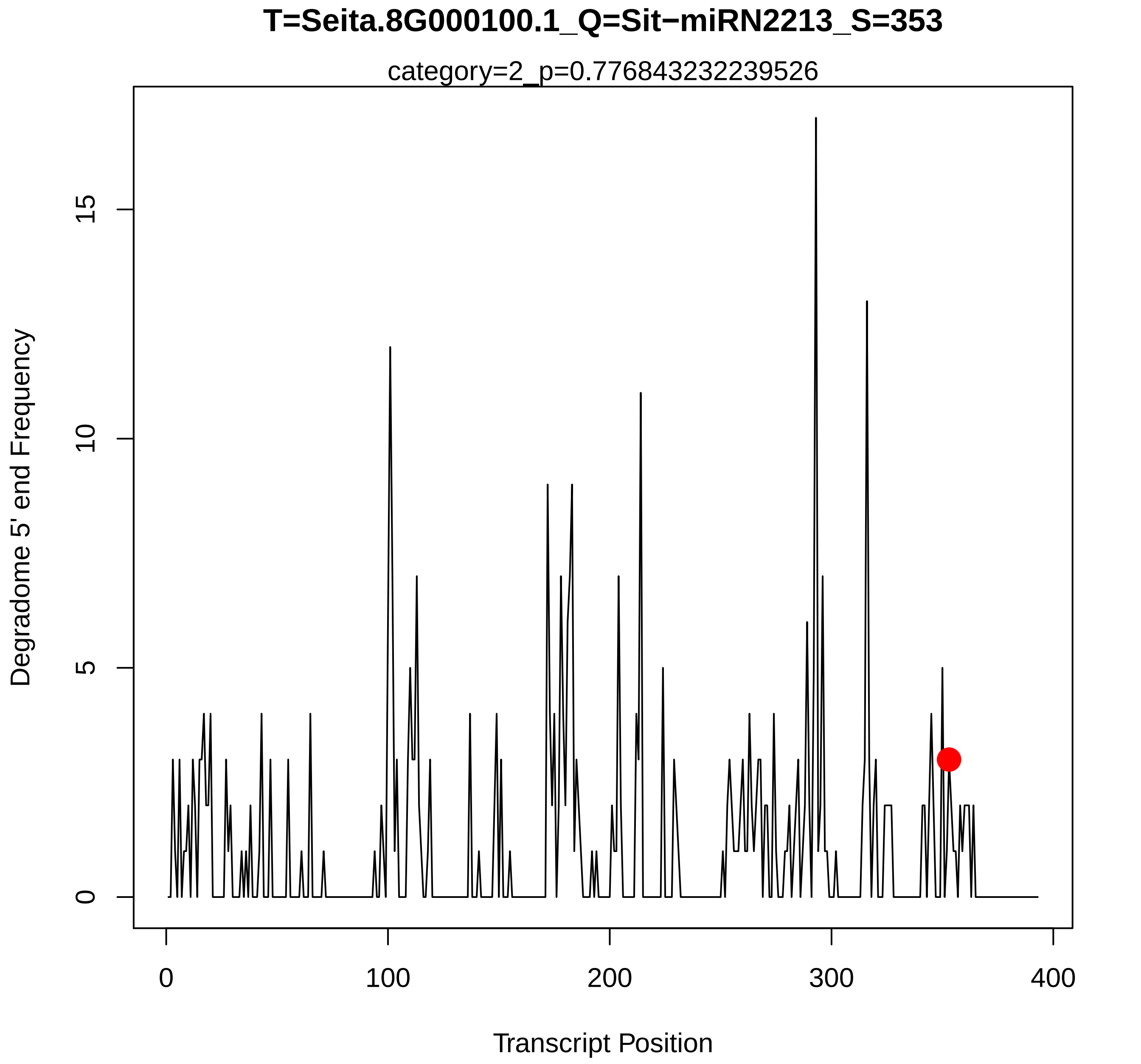
T=Seita.8G000100.1_Q=Sit−miRN2213_S=353
category=2_p=0.776843232239526
Degradome 5' end Frequency
0
5
10
15
0
100
200
300
400
Transcript Position
